# Metronomic capecitabine vs. best supportive care in Child-Pugh B hepatocellular carcinoma: a proof of concept

**DOI:** 10.1038/s41598-018-28337-6

**Published:** 2018-07-03

**Authors:** Stefania De Lorenzo, Francesco Tovoli, Maria Aurelia Barbera, Francesca Garuti, Andrea Palloni, Giorgio Frega, Ingrid Garajovà, Alessandro Rizzo, Franco Trevisani, Giovanni Brandi

**Affiliations:** 1 Oncology Unit, Department of Experimental, Diagnostic and Specialty Medicine, Sant’Orsola-Malpighi Hospital, University of Bologna, Bologna, Italy; 2 Internal Medicine Unit, Department of Medical and Surgical Sciences, Sant’Orsola-Malpighi Hospital, University of Bologna, Bologna, Italy; 3 Medical Semeiotics Unit, Department of Medical and Surgical Sciences, Sant’Orsola-Malpighi Hospital, University of Bologna, Bologna, Italy; 4 “G.Prodi” Interdepartmental Centre for Cancer Research, University of Bologna, Bologna, Italy

## Abstract

There is a relative lack of evidence about systemic treatments in patients with hepatocellular carcinoma (HCC) and moderate liver dysfunction (Child-Pugh B). In this multicenter study we retrospectively analyzed data from Child-Pugh B-HCC patients naïve to systemic therapies, treated with MC or best supportive care (BSC). To reduce the risk of selection bias, an inverse probability of treatment weighting approach was adopted. Propensity score was generated including: extrahepatic spread; macrovascular invasion; performance status, alphafetoprotein > 400 ng/ml, Child- Pugh score [B7 vs. B8–9]. We identified 35 MC-treated patients and 70 controls. Median overall survival was 7.5 [95% CI: 3.733–11.267]in MC-patients and 5.1 months [95% CI: 4.098–6.102] in the BSC group (p = 0.013). In patients treated with MC, median progression-free survival was 4.5 months (95% CI: 2.5–6.5). The univariate unweighted Cox regression showed a 42% reduction in death risk for patients on MC (95%CI: 0.370–0.906; p = 0.017). After weighting for potential confounders, death risk remained essentially unaltered. In the MC group, 12 patients (34.3%) experienced at least one adverse event, the most common of which were: fatigue (17.1%), hand-foot syndrome (8.5%), thrombocytopenia (8.5%), and neutropenia (5.7%). MC seems a safe option for Child-Pugh B-HCC patients. Its potential antitumour activity warrants prospective evaluations.

## Introduction

Patients with hepatocellular carcinoma (HCC) and moderately compromised liver function (Child-Pugh B functional status) have limited therapeutic options due to the risk of iatrogenic liver failure. Furthermore, the risk-benefit ratio of available treatments may be unfavorable, because of the concurrent risk of non cancer-related deaths deriving from progressive hepatic failure^1^.

This is particularly evident in the HCC cases not amenable to surgery or locoregional treatments. For these patients, the availability of sorafenib depends on the different international guidelines and local regulatory agencies policies^2^. For instance, the Japanese Society of Hepatology guidelines consider Child Pugh B (CP-B) HCC as compromised patients for whom best supportive care (BSC) is recommended^3^. Instead guidelines by the Asia-Pacific Society for Hepatology, National Comprehensive Cancer Network, European Association for the Study of the Liver, and American Association for the Study of Liver Diseases are more permissive and suggest a possible use of sorafenib^4–6^. Moreover, regulatory agencies of different countries limit the prescription of sorafenib only in CP-A patients (including Australia, Belgium, Canada, France, Italy, Netherlands, South Korea, Switzerland and Taiwan)^1^. This discrepancy is justified by the relative lack of clinical data of sorafenib in CP-B HCC. Only few CP-B patients were included in the registrative SHARP and Asian-Pacific Phase III trials to minimize the confounding effect of non cancer-related death^7,8^. An investigator-initiated RCT was specifically designed to assess the risk/benefit ratio of sorafenib in CP-B patients, but, unfortunately, it was prematurely interrupted (ClinicalTrial.gov Identifier: NCT01405573)^9^. More information came from the GIDEON (Global Investigation of therapeutic DEcisions in HCC and Of its treatment with sorafeNib) trial. This large Phase 4 study evaluated the safety and the efficacy of sorafenib in HCC patients under real-life practice conditions, and also in several clinically relevant subgroups, including patients with CP-B liver function^10^. Median overall survival (OS) was longer in CP-A patients than in CP-B (13.6 months vs. 5.2 months, respectively). The poor OS, paired with an higher rate of serious adverse events (AEs) compared to the more compensated patients, questioned the overall risk/benefits balance^10^. Previous reports^11–13^ had also reached similar conclusions.

It is also unlikely that CP-B patient may benefit from the new agents which emerged as successful systemic treatments for HCC, namely lenvatinib^14^, regorafenib^15^, and cabozantinib^16^. Patients with compromised liver failure were not included in these trials for the same reasons they were excluded from the SHARP trial. Furthermore, the rate and the severity of toxicities of these new anti-VEGFR agents are widely overlapping with those of sorafenib. It is therefore likely that most regulatory agencies will not approve these new drugs in CP-B subjects. At the moment, only a dedicated cohort of the CheckMate-040 nivolumab ongoing study^17^ has the specific aim of evaluating the efficacy and the safety of an anticancer agent in CP-B patients.

In recent years, the concept of metronomic chemotherapy has been introduced into oncology^18^. It is based on the chronic administration of chemotherapeutic agents at low doses without prolonged drug-free breaks to optimise the antiangiogenic properties of the drug and to reduce toxicities^19,20^.

Metronomic capecitabine (MC) has been tested as in first and in second line treatment for HCC patients by several studies, which demonstrated a good anticancer activity as well a very low rate of toxicities^21–24^. Also, MC may be effective and well tolerated in recurrent HCC after liver transplantation^25^. Nevertheless, none of these studies provided information about the potential effect of MC in CP-B HCC patients. The aim of this study was to retrospectively evaluate the safety and efficacy of MC as first-line treatment in this class of HCC patients.

## Results

A total of 105 consecutive patients were included in our study: 35 were treated with MC (cases) from April 2006 to February 2017, while 70 received BSC alone (controls) from October 2004 to October 2015.

Among the study population, a total of 58 patients (55.2%) were allocated to either MC or BSC in the 2006–2011 timelapse. Of these patients, 16 received MC and 42 BSC (45.7% vs. 60.0% of the overall recruitment of the respective study group, p = 0.212).

Among the MC patients, 27 subjects (77%) were male, with a median age of 65 years (range 43–88). Instead, in the BSC group, 57 patients (81.4%) were males, with a median age of 69 years (range 37–91).

Characteristics of the two groups have been reported in Table 1. After application of the propensity score, the standardized differences between groups was generally minor, which suggested that baseline characteristics were equal (Table 2).

**Table 1:** Baseline characteristics of patients.

Variables	MC n (%)	BSC n (%)	P values
Total	35	70	
Sex			
Male	27 (77%)	57 (81.4%)	0.613
Female	8 (23%)	13 (18.6%)	
Age median (range)	65 (43–88)	69 (37–91)	0.136
BCLC			
B	15 (42.8%)	30 (42.8%)	1.000
C	20 (57.2%)	40 (57.2%)	
Extra-hepatic spread	9 (25.7%)	12 (17.1%)	0.312
Macrovascular Invasion	14 (40%)	29 (41.4%)	1.000
ECOG - PS			
0	23 (65.7%)	39 (55.6%)	0.401
1–2	12 (34.3%)	31 (44.4%)	
Child-Pugh score			
B7	18 (51.4%)	43 (61.4%)	0.402
B8-B9	17 (48.6%)	27 (38.6%)	
AFP > 400 ng/mL	11 (31.4%)	20 (30.0%)	1.000
Etiology			
HBV	6 (17.1%)	8 (11.4%)	
HCV	18 (51.4%)	37 (52.8%)	0.703
Non-viral	11 (31.4%)	25 (35.7%)

**Table 2:** Checking balance of confounders between MC and BSC group after weighting.

	Mean in MC	Mean in BSC	Standardized differences
Sex	0.77	0.81	−0.095
Age	65.34	68.39	−0.308
Etiology (*Viral vs*. *Non Viral*)	0.72	0.74	−0.091
Child-Pugh score (*B7 vs*. *B8–9*)	0.49	0.39	0.201
BCLC	0.57	0.57	0
ECOG-PS	0.34	0.44	−0.202
Extrahepatic spread	0.26	0.17	0.236
Macrovascular Invasion	0.4	0.41	−0.02
AFP > 400 ng/mL	0.31	0.3	0.021

*MC* = Metronomic capecitabine; BSC = Best supportive care; BCLC = Barcelona Clinic Liver Cancer; ECOG-PS = Eastern Cooperative Oncology Group-Performance Status; AFP = Alpha-fetoprotein.

**Figure 1:**
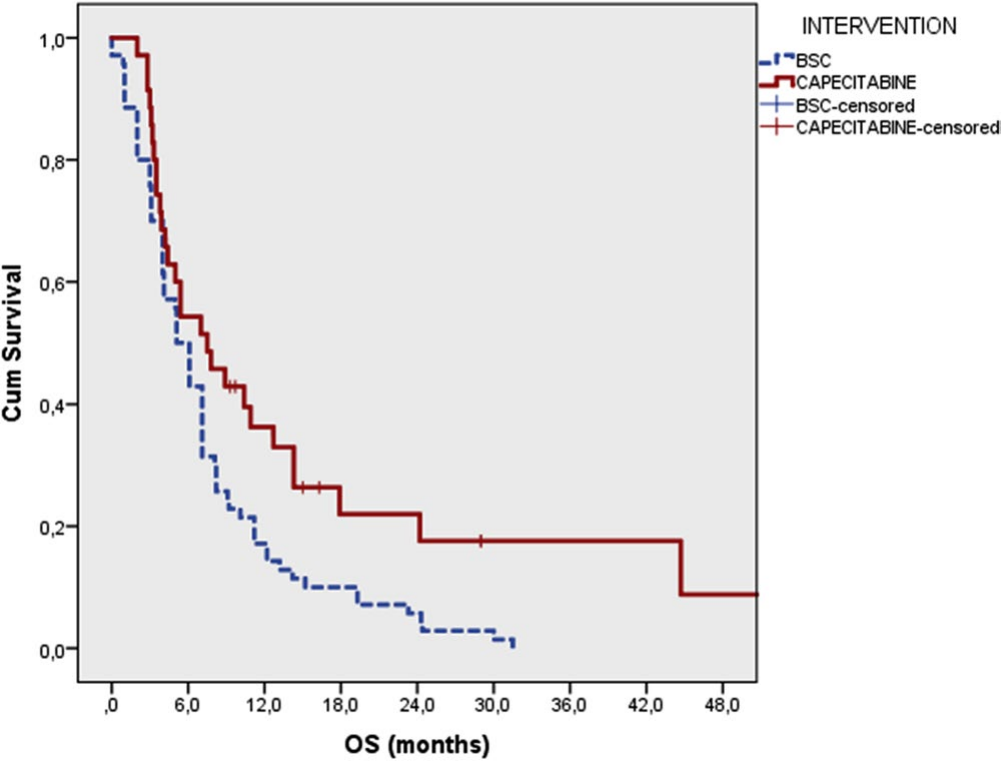
Overall survival of patients treated with metronomic capecitabine and best supportive care.

**Figure 2:**
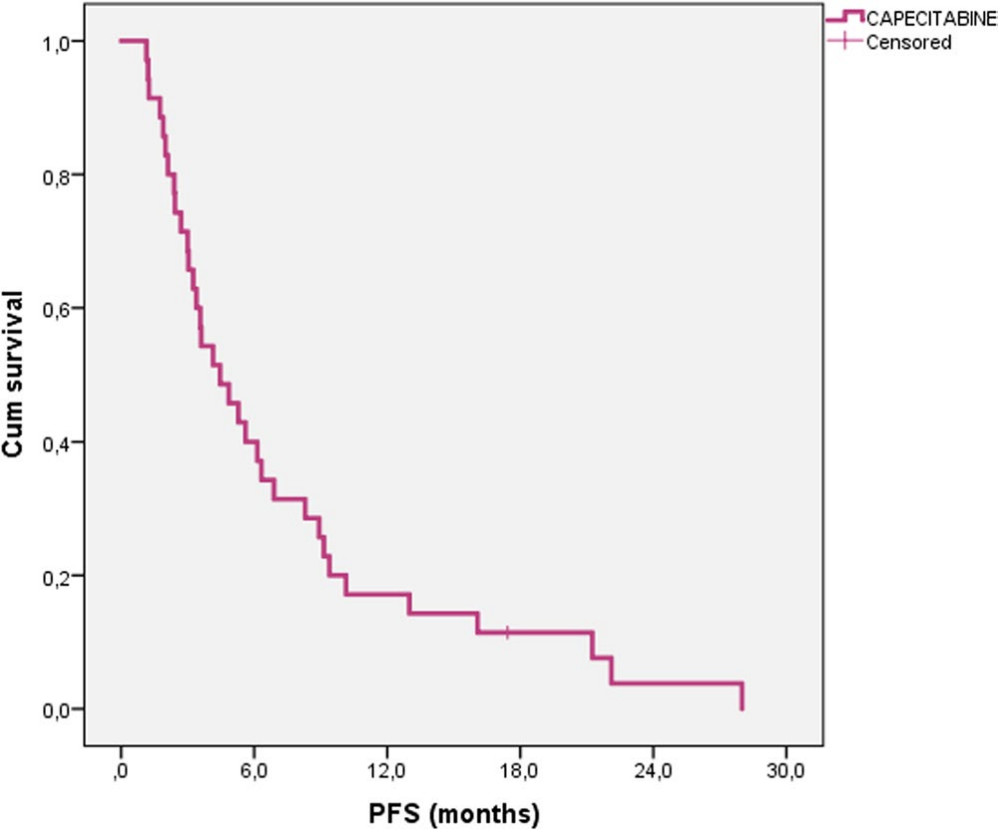
Progression free survival of patients treated with metronomic capecitabine.

Median follow-up was 6 months (1.1–52.0). Median OS was 7.5 months [95% CI: 3.733–11.267]in patients receiving MC and 5.1 months [95% CI: 4.098–6.102] in patients receiving BSC (p = 0.013) (Fig. 1). The 1-year and 2-year survival rate were 31.4 vs. 17.1% and 14.3 vs. 5.7% in the MC and BSC groups, respectively, In patients treated with MC, median PFS was 4.5 months (95% CI: 2.5–6.5) (Fig. 2), with a median treatment duration of 4.8 months (1.1–50.1).

The result of univariate unweighted Cox regression model showed a 42% reduction in death risk for patients on MC (HR 0.579; 95%CI: 0.370–0.906; p = 0.017) (Table 3). After weighting for potential confounders, death risk remained essentially unaltered (HR 0.525; 95%CI: 0.332–0.829; p = 0.006).

**Table 3:** Univariate and multivariate analysis of the correlation between OS and the analyzed independent variables.

Variable	Univariate		Multivariate	
	Median OS (95% CI)	p-value	Hazard Ratio OS (95% CI)	p-value
Gender				
Male	9.1 (6.1–12.1)	0.100		
Female	5.1 (3.9–6.3)			
Etiology				
Viral	6.1 (4.9–7.3)	0.309		
Non-Viral	5.1 (3.8–6.4)			
Era of enrollment				
2006–2011 (0)	6.1 (4.1–8.1)	0.960		
2012–2017 (1)	5.0 (2.2–7.8)			
Child-Pugh				
B7	6.1 (4.7–7.5)	0.816		
B8-B9	5.1 (3.3–6.9)			
BCLC				
B	7.8 (6.3–9.3)	0.092	*Excluded for co-linearity with macrovascular invasion and extra-hepatic spread	
C	5.0 (3.6–6.4)			
ECOG-PS				
0	6.1 (4.8–7.5)	0.533		
1–2	5.4 (4.0–6.8)			
Macrovascular invasion				
Absent	7.1 (5.5–8.7)	**0**.**042**	1.336 (0.876–2.040)	**0**.**179**
Present	5.0 (3.8–6.2)			
Extra-hepatic spread				
Absent	7.0 (5.9–8.1)	0.058	1.950 (1.135–3.350)	**0**.**016**
Present	3.1 (2.9–3.3)			
AFP (ng/mL)				
<400	6.1 (4.5–7.7)	0.151		
>400	5.4 (3.0–7.8)			
Treatment group				
MC	7.5 (3.7–11–3)	**0**.**013**	0.525 (0.332–0.829)	0.006
BSC	5.1 (4.1–6.1)			

*BCLC* = Barcellona Clinic Liver Cancer; ECOG-PS = Eastern Cooperative Oncology Group-Performance Status; AFP = Alpha-fetoprotein; MC = Metronomic capecitabine; BSC = Best supportive care.

Best tumor response in patients treated with MC was partial response in 3 patients (8.6%), stable disease in 10 patients (28.6%) and progressive disease in 22 patients (62.9%). No complete response was observed. Disease control (defined as partial response + stable disease) was associated with a significantly better survival compared to progressive disease (24.2 vs. 3.9 months, p < 0.001).

In the MC group, 12 patents (34.3%) experienced at least one AE, the most common of which were: fatigue (17.1%), hand-foot syndrome (8.5%), thrombocytopenia (8.5%), neutropenia (5.7%), anaemia (2.9%), bilirubin elevation (2.9%), nausea/vomiting (2.9%), mucositis (2.9%) (Table 4).

**Table 4:** Adverse events of MC categorized according to the National Cancer Institute Common Terminology Criteria for Adverse Events (CTCAE) version 4.

	Any grade N patients (%)	Grade 1–2 N patients (%)	Grade 3–4N patients (%)
Overall	12 (34.3%)	8 (22.8%)	2 (5.7%)
Fatigue	6 (17.1%)	6 (17.1%)	0
Hand-Foot skin reaction	3 (8.5%)	2 (5.7%)	1 (2.9%)
Thrombocytopenia	3 (8.5%)	1 (2.9%)	2 (5.7%)
Neutropenia	2 (5.7%)	2 (5.7%)	0
Anaemia	1 (2.9%)	1 (2.9%)	0
Bilirubin elevation	1 (2.9%)	1 (2.9%)	0
Nausea/Vomiting	1 (2.9%)	1 (2.9%)	0
Mucositis	1 (2.9%)	1 (2.9%)	0

Although our study was not specifically designed to assess a correlation between dermatological AEs and tumor response, it should be noted that all of the 3 patients with treatment-emergent hand-foot syndrome attained disease control as their best radiological response (2 stable disease and 1 partial response). Further, all of these patients were alive after 1 year of treatment (with an OS of 29.0, 15.0 and 12.7 months, respectively).

## Discussion

No systemic treatment is universally accepted for CP-B HCC. In this study we provided a proof of concept for future trials of MC.

As a first point, we demonstrated an improved OS in comparison with matched patients treated with BSC alone. The potential mechanisms of actions of MC have been previously illustrated^26^ and include: inhibition of tumor angiogenesis, reduction of the therapeutic resistance of the tumor, and activation of the adaptive and innate immune response^27,28^.

Second, we described a low rate of AEs in this fragile setting. In particular, G3-G4 toxicities occurred in slightly more than 5% of the patients and no severe AEs were reported. These favorable profile had been previously reported in compensated patients^21–24^, but safety data in CP-B patients were still lacking. The continuous subministration of low, fractioned, doses of drugs characterizing the metronomic regimens was the key element in determinating the good tolerability of MC^29^. It should not be disregarded that MC is a relatively inexpensive treatment (in Italy it costs less than 100 euro/month), representing a therapeutic approach easily affordable by most National Health Systems.

In comparison with previous studies of MC in compensated patients, the OS was unsurprisingly lower. In particular, in a post-sorafenib setting, Casadei-Gardini *et al*. described a median OS of 12 months^23^, while Trevisani *et al*. reported median survival of 9.5 months in a larger cohort^24^. Similarly, the median OS was 14.5 months in pivotal study by Brandi *et al*.^21^. Interestingly, the reduction in the risk of mortality witnessed in our study was not substantially different from that described in CP-A patients^23,24^. Even more interestingly, 37% of our patients achieved a best radiological response of disease control, which was associated with a significantly longer survival. As a result, 31.4 and 14.3% of patients in the MC treatment arm were alive at 12 and 24 months, underlying the possibility of “long survivors” even in this difficult clinical setting.

In the absence of head-to-head trials, a comparison with studies of sorafenib in the CP-B population is more difficult and potentially misleading. CT-B patients were not included in the registrative SHARP and Asian-Pacific trials, however a number of retrospective and prospective trials described the performance of sorafenib in CP-B patients (reviewed in^1^). Data of efficacy are quite heterogeneous, with median OS values ranging from 3.2^30^ to about 8.0 months^31,32^. It should be underlined that the prevalence of AEs, although similar to that recorded in CP-A patients was still high. In particular, G3-G4 toxicities have been described in 30–32% of CP-B subjects^10,33^. Furthermore, severe AEs were more frequent in CP-B than in CP-A patients^10^.

As a result, median treatment duration never exceeded 2.5 months^10,11,30,34–36^. In comparison to these data, our study suggest that MC may result more easily tolerated, thus allowing a longer treatment duration (which, in its turn, might lead to a sustained response in a subset of patients).

Our study has some limitations, due in particular to its retrospective nature. However, it should be underlined that cases were consecutively selected, thus reducing potential biases. Moreover, lack of randomization was limited applying propensity score to the most common determinants of survival of HCC patients receiving systemic treatment. Finally, the results from the Cox regression models, remained substantially unaltered after weighting for potential confounders. The long timeframe of the study was another potential confounder. The treatment for underlying HVB infection saw its latest major innovation before the initial date of recruitment, with the licensing of entecavir and tenofovir in 2004 and 2015, respectively. Instead, new direct antiviral agents became available for the treatment of HCV infection in 2012 and 2013. To address this potential confounder, the era of recruitment was considered as a variable in the survival analysis. No correlation was found between era and OS.

In conclusion, this study provides a proof-of-concept of the efficacy and safety of MC in CP-B patients. Our results may be of help in the identification of a possible treatment for CP-B patients who can not be prescribed with sorafenib due to regulatory restrictions. Similarly, MC could represent a therapeutic alternative for CP-B patients intolerant to tyrosine-kinase inhibitors.

These promising results need confirmation through prospective RCTs.

## Materials and Methods

### Patients and Treatment

In this multicentre study, we retrospectively analyzed data of CP-B HCC patients naïve to systemic therapies, not suitable for surgical resection, liver transplantation, percutaneous ablation procedures or endovascular treatments. These patients were treated with either MC or BSC alone, as sorafenib prescription is not allowed for CP-B patients by the regulatory agency of our Country.

Patients with any of the following exclusion criteria were considered not eligible for receiving MC: a) Eastern Cooperative Oncology Group performance status score (ECOG PS) > 2; b) severe ascites; c) moderate-to-severe encephalopathy; d) bilirubin ≥ 3 mg/dL; c) creatinine ≥ 1.5 mg/dL; d) platelets < 30000/mmc; e) neutrophils < 1000/mmc; f) peripheral neuropathy; g) symptomatic congestive heart failure, unstable angina pectoris, or serious cardiac arrhythmias. For the purposes of this study, we excluded from the analyses of the BSC group all of the patients who had any of the aforementioned exclusion criteria.

Patients received MC if its prescription was allowed by the centers policy and patients accepted this off-label treatment after receiving adequate information and signing an informed consent. Eligible patients received oral capecitabine at the metronomic dosage of 500 mg every 12 hours. The treatment was continued until the occurrence of unacceptable toxicity or radiological or symptomatic progression of HCC. No addition surgical, loco-regional or systemic treatments were performed during MC treatment. Patients undergoing further treatments after MC permanent withdrawal (n = 2: one case of wedge resection for HCC at high risk of rupture and one case of trans-arterial embolization for intra-tumor bleeding) were censored at the time of the treatment.

AEs and laboratory abnormalities were graded using the National Cancer Institute Common Terminology Criteria for Adverse Events (CTCAE) version 4. Drug-related AEs were managed with supportive therapy, dose reduction or treatment suspension.

Patients were treated with BSC alone if: a) they were eligible for MC but refused this option or b) if MC prescription was not contemplated by the centres policies. In particular, the Oncology Unit began switching patients to MC in 2006, and the Internal Medicine Unit in 2010. Both groups underwent laboratory and clinical follow-up monthly. Radiological examination was repeated every 2–3 months in MC patients, using multiphase computed tomography (CT) or magnetic resonance imaging (MRI). In BSC controls, imaging procedures were performed only when considered clinically necessary. Tumor response to MC was evaluated by modified Response Evaluation Criteria In Solid Tumor (mRECIST)^37^.

All patients provided written informed consent and the study was approved by the Independent Ethics Committee of the Bologna Authority Hospital S.Orsola-Malpighi. All methods were performed in accordance with the relevant guidelines and regulations.

### Statistical analysis

Categorical variables were expressed as frequencies. Continuous variables were presented as median (range). OS was defined as the time from start date of the treatment to date of death. Progression-free survival (PFS) was defined as the time form start date of MC to the date of progression or death or last follow up, whichever occurred first. OS and PFS were reported as median values and expressed in months, with 95% confidence intervals (CI). Survival curves were estimated using the product-limit method of Kaplan-Meier. The role of stratification factors was analyzed with log-rank tests. Propensity score is the conditional probability of being treated given a set of observed potential confounders. In this way, all the information from a group of potential confounders is summarized into a single balancing score variable. Propensity score assures that the distribution of measured baseline covariates is maintained unchanged in treated and untreated subjects. Standardized differences were used as balance measure to compare the difference in means in unit of the pooled standard deviation.

A weighted Cox Proportional Hazard Model was performed including treatment with capecitabine as covariate where all confounding factors has been controlled by weighting. Propensity score weights were computed as 1/propensity score for patients treated with MC and 1/(1-propensity score) for patients treated with BSC. P-values < 0.05 were considered statistical significant. Considering the long timelapse of this study, an analysis of the era of enrolment (2006–2011 vs 2012–2017) was performed to reduce the risk of confounding factors.

Statistical analysis were performed using SPSS version 23.0 (SPSS Inc., Chicago, IL, USA).

### Data availability

The datasets generated during and/or analysed during the current study are available from the corresponding author on reasonable request.
